# Tert-Butanol as a Structuring Agent for Cellulose Nanocrystal Fluids and Foams

**DOI:** 10.1021/acs.biomac.5c00184

**Published:** 2025-07-18

**Authors:** Saül Llácer Navarro, Elliot Orzan, Ratchawit Janewithayapun, Paavo Penttilä, John Andersson, Anna Ström, Roland Kádár, Tiina Nypelö

**Affiliations:** † Department of Chemistry and Chemical Engineering, 11248 Chalmers University of Technology, 41296 Gothenburg, Sweden; ‡ Wallenberg Wood Science Center, Chalmers University of Technology, 41296 Gothenburg, Sweden; § Department of Bioproducts and Biosystems, 211731 Aalto University, 02150 Espoo, Finland; ∥ Department of Industrial and Materials Science, Chalmers University of Technology, 41296 Gothenburg, Sweden

## Abstract

Nanocelluloses are uniquely valued for their high surface area and controllable assembly. This study elucidates the assembly of cellulose nanocrystals (CNCs) in tert-butanol (TBA) and water mixtures. We emphasize the influence of TBA on the structure of suspensions and freeze-dried foams. Although the length-scale of CNC organization is large relative to water-TBA structures, adding more than 30 wt % TBA shifted ordered CNC packing into an isotropic network. The change was attributed to the disruption of ionic interactions and adsorption of TBA to hydrophobic CNC interfaces; manifesting as a 5-fold increase in viscosity at 50 wt % TBA content. The freeze-dried foams’ morphology was transformed due to TBA-modulated crystal growth during the freezing process. This led to the intriguing capability to control foams’ mechanical strength and surface area, achieving up to 3 and 15-fold increases, respectively. The investigations highlight TBA’s potential as a structuring agent in solvent-mediated design of nanomaterial systems.

## Graphical abstract

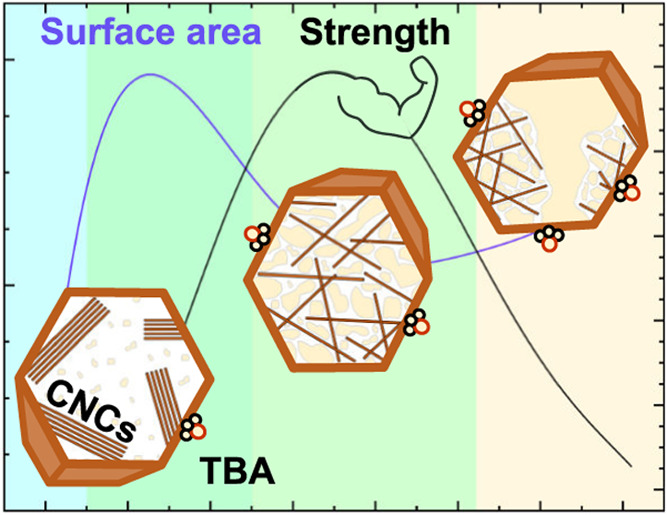

## Introduction

The use of alcohol–water mixtures to change the physical and chemical properties of material systems has fascinated scientists for centuries. Tert-butanol (TBA) is a particularly intriguing monohydric alcohol as it has a high freezing point of 25.5 °C and is soluble in water despite its particular amphiphilicity and size. The addition of TBA to water controls the local density, intermolecular interactions, and dielectric constant of the TBA-water system.^1,2^ Dynamic TBA-rich and water-rich regions are formed at the molecular level as TBA disrupts the hydrogen bonding between water molecules.^3–8^ This phenomenon is most pronounced at TBA concentrations between 30 and 50 wt %, which leads to the formation of inhomogeneities at the submicron scale.^3–5^ The hydrophobic effect, arising from the presence of methyl groups on TBA, generates discrete TBA-rich regions dispersed in the continuous aqueous phase.^4^ The structuring of these alcohol–water systems has been used in nanotechnology to e.g., control the porosity of aerogels.^9^

Cellulose is a critical component in the development of materials science and engineering solutions, as it is a renewable product abundantly available from plants. The discovery of cellulose nanomaterials has opened an avenue for the merging of cellulosic materials with nanotechnology.^10^ Cellulose nanocrystals (CNCs) are nanoparticles that form colloidal suspensions and gels in water. In dilute suspensions, CNCs are present as a dispersed isotropic phase. However, above a critical concentration in water (>3 wt %), entropic forces cause CNCs to pack and spontaneously self-assemble.^11–15^ CNC assembly and the gelation of the system can be modified by adjusting the ionic strength using salts,^16–19^ and by introducing cosolvents such as glycerol and alcohols.^20,21^

The molecular structuring of alcohol–water mixtures only spans a few ångströms, while nanocelluloses are magnitudes larger in size. It is not straightforward to accept that molecular level structuring could mobilize nanocelluloses and guide their assembly. Nonetheless, several mechanisms have been proposed as the driving forces behind the structuring of nanocelluloses in alcohol–water mixtures. The inclusion of TBA is thought to disrupt interfibril hydrogen bonding between cellulose as the three bulky methyl groups on TBA molecules introduce steric hindrance.^22–25^ Moreover, the effective range of electrostatic interactions between charged CNCs also diminishes as a result of the addition of TBA, resulting in a reduction of the Debye length.^26,27^ A decrease in the Debye length can lead to reduced repulsive forces, potentially causing destabilization and particle aggregation.

The arrangement of nanocelluloses in suspensions, such as CNCs, is thought to direct their assembly in solid materials such as nanopapers, aerogels, and cryogels.^22–25,28–31^ This study hypothesizes that the incorporation of TBA into CNC-water suspensions disrupts the nanoscale to macro-scale self-assembly of CNCs in both suspensions and their subsequent freeze-dried foams. The nanoscale assembly of CNC suspensions was investigated using small-angle X-ray scattering (SAXS) and wide-angle X-ray scattering (WAXS) and rheology was investigated to understand the macro-scale behavior. Furthermore, the influence of TBA on crystal growth during freezing to dry foams was assessed via scanning electron microscopy (SEM), mechanical testing, and surface area determination. Elucidating the driving forces behind nanoparticle assembly in alcohol–water systems can provide a powerful technique for intentionally structuring amphiphilic particles.

## Experimental Section

## Materials

CNCs were purchased from Celluforce (Canada). TBA and HCl were acquired from Sigma-Aldrich (Sweden). Milli-Q water (resistivity 18.2 Ω^–1^ cm^–1^ at 25 °C) was obtained via a Millipore water purification system. A 10 wt % CNC stock suspension was prepared in Milli-Q water and stirred using IKA EUROESTAR 60 (Germany) overhead stirrer with a four-bladed R1342 propeller at 1200 rpm for 1 h.

## Methods

### Design of Experiments

The experiments were designed using JMP Pro 16.2.0 software from SAS. The software established compositions of ternary suspensions containing water, TBA, and CNCs (Supporting Information: Composition ternary plot, Figure S1). The accessible range was limited by the concentration of the 10 wt % CNC stock suspension in water. The following naming convention was applied: xCNCyT, where *x* is the concentration of CNC in wt %, and *y* is the concentration of TBA in wt %.

### Preparation of CNC Suspensions

The suspensions were prepared by diluting the CNC stock suspension with Milli-Q water. TBA was then added in seven incremental steps with thorough shaking after each step to avoid phase-separation. The suspensions were shaken overnight on a moving platform at 180 rpm.

### Small-Angle and Wide-Angle X-Ray Scattering

Small-angle X-ray scattering (SAXS) and wide-angle X-ray scattering (WAXS) were measured using a SAXSLAB Mat: Nordic (Denmark) bench-scale instrument, with Cu Kα radiation and a *q* (scattering vector magnitude) range of 0.003–2.7 Å^–1^. Detection was done with a Pilatus 300k detector with sample-to-detector distances of 1530 mm for SAXS and 130 mm for WAXS. The beam size for the SAXS configuration was 0.2 mm. *q*-calibration was performed using LaB_6_ powder. The suspensions were tested at room temperature in glass capillaries of 2 mm in diameter (WJM-Glas, Germany) and 0.01 mm wall thickness. Transmission correction was applied to express the intensity as absolute units × thickness. The masking of the beam stop area and the gaps between the detector elements was performed by SAXSGUI software (version 2.27.03), the scattering from an empty capillary was subtracted in MATLAB, and model fitting was performed in SasView software (version 5.0.5). For 1D data, azimuthal averaging was performed for all angles to obtain the scattering intensity as a function of *q*.

### Quartz Crystal Microbalance with Dissipation Monitoring

For conducting QCM-D measurements, a Q-Sense E4 apparatus (Biolin Scientific) was employed. Frequencies of the third, fifth, and seventh overtones were monitored on 5 MHz silicon dioxide sensor crystals. The sensors underwent spin coating twice at 4000 rpm using a SPIN 150 (SPS-Europe, Netherlands) device with 100 μL of a 1 wt % CNC suspension, followed by drying overnight at 80 °C. The QCM-D temperature was maintained at 25 °C, and the flow rate was 150 μL/min. Responses were calculated as the net difference from an uncoated reference sensor and baseline-corrected relative to water injections. The alcohol blends utilized were at a concentration of 12 wt %.

### Surface Plasmon Resonance Spectroscopy

SPR measurements were carried out using a Bionavis 220A Navi multiparameter SPR device with silicon dioxide coated gold sensors. A diode wavelength of 670 nm was continuously scanned between 58.0° and 77.9° for a duration of 3.88 s per scan. Liquids were introduced at a 20 μL/min, with the system temperature set at 25 °C. Resonance shifts were corrected for bulk effects as described by eq 5 in Svirelis et al.^32^ (using the same parameters for *S*_*SPR*_, *S*_*TIR*_ and δ, with an estimated film thickness of *d* = 10 nm).

### Rheological Characterization

The rheological properties of the suspensions were determined using a TA Discovery HR-3 rheometer, TA Instruments. A 40 mm diameter sandblasted plate with a measuring gap of 1 mm was used for suspensions containing CNCs. For systems consisting only of water and TBA, a 40 mm cone with a truncation angle of 1° and a preset measuring gap of 26 μm was used. During the measurements, a Peltier lower plate was used to maintain a temperature of 20 °C. An equilibration time of 300 s was applied before the measurements were made. The shear viscosity was measured at shear rates of 10–1000 s^–1^ (for solutions without CNCs) and 0.015–1000 s^–1^ (suspensions containing CNCs). Shear strain sweep measurements were conducted at 1 Hz and a strain amplitude of 0.01%–800%. All rheological data obtained were analyzed in the TRIOS software.

### Ternary Plot Models

Experimental values were fitted using a mixture of surface response and a second-degree polynomial in the JMP software. Step-wise fitting was used to find the model with the most significant effects. The fitted models provided an easier visualization of the trends in the customized ternary plots. The adjusted R-squared value was at least 0.9.

### Freeze-Drying of the Suspensions Into Solid Foams

The suspensions were injected into cylindrical molds with a diameter of 1 cm and a height of 2 cm, followed by degassing. The molds had particularly thick walls, a thin bottom plate and top. The molds were subsequently submerged in liquid nitrogen and placed in a ScanVac CoolSafe freeze-dryer at −105 °C for 2 days until they developed into free-standing dry foams.

### Density and Porosity of the Solid Foams

The envelope density (ρ_E_) of solid foams was measured using calipers and a high-precision balance. Skeletal density (ρ_S_) was measured in a Micromeritics AccuPyc II gas displacement pycnometer using He gas. Relative density, ρ_R_, was given as the ratio of envelope density to skeletal density (eq 1). The porosity was then calculated using eq 2 according to Gibson.^33^

1 $$\rho_{R}=\frac{\rho_{E}}{\rho_{S}}$$

2 $$P\;\left(\%\right)=\left(1-\rho_{R}\right)\times 100$$

### Determination of Solid Foam Surface Area

The surface area was quantified using the Brunauer–Emmett–Teller (BET) method, employing a Micrometrics TriStar 3000 analyzer with TriStar II Plus software. Nitrogen (N_2_) gas was utilized within a relative pressure range of 0–0.98. For BET linearization, the relative pressure corresponding to the nitrogen monolayer coefficient in the BET equation was included and the maximum value of the Rouquerol BET graph was the last chosen point.

### SEM Imaging of Solid Foams

The solid foams were cut through the center axially with a fresh razor to maintain the internal structure and sputter-coated with 4 nm of gold. Visualization was performed on a JEOL 7800F Prime instrument at an acceleration voltage of 5 kV.

### Mechanical Testing of Solid Foams

Solid foams were conditioned at 20 °C in a desiccator for a week prior to mechanical testing. Compression tests were performed using the texture analyzer (Stable Microsystems, UK) at a rate of 1 mm s^–1^ after reaching a preload force of 0.5 N. The cylindrical solid foams were pressed parallel to the axial direction between 40 mm diameter parallel plates using a 50 kg load cell. The compressive modulus was taken as the slope of the stress–strain curve below 10% strain, while the compressive strength corresponded to the stress at 10% strain. Energy absorption was calculated as the area under the stress–strain curve up to 50% strain. Variations in density between solid foams were accounted for by dividing all values by their relative density.

## Results

### Submicron Arrangement of CNCs

#### X-ray Scattering of CNC Suspensions

The presence of CNCs without any TBA (5CNC0T) led to the appearance of the 200 diffraction peak of cellulose I_β_ around *q* = 1.6 Å^–1^ and the 11̅0 and 110 peaks between *q* = 1.0 and 1.2 Å^–1^ in the WAXS diffractogram (5CNC0T in Figure 1a). However, these cellulose peaks at *q* = 1.0 and 1.2 Å^–1^ were difficult to distinguish from the scattering of the hydrophobic interactions between aliphatic TBA groups (1.25 Å^–1^) at TBA concentrations of 30 and 46 wt % (5CNC30T and 5CNC46T).^3^ Moreover, while it is known that TBA forms structures in water, TBA-water structuring was present even after the addition of CNCs (Figure 1a vs Supporting Information: WAXS of 0 wt % CNCs with TBA, Figure S2).

**Figure 1:**
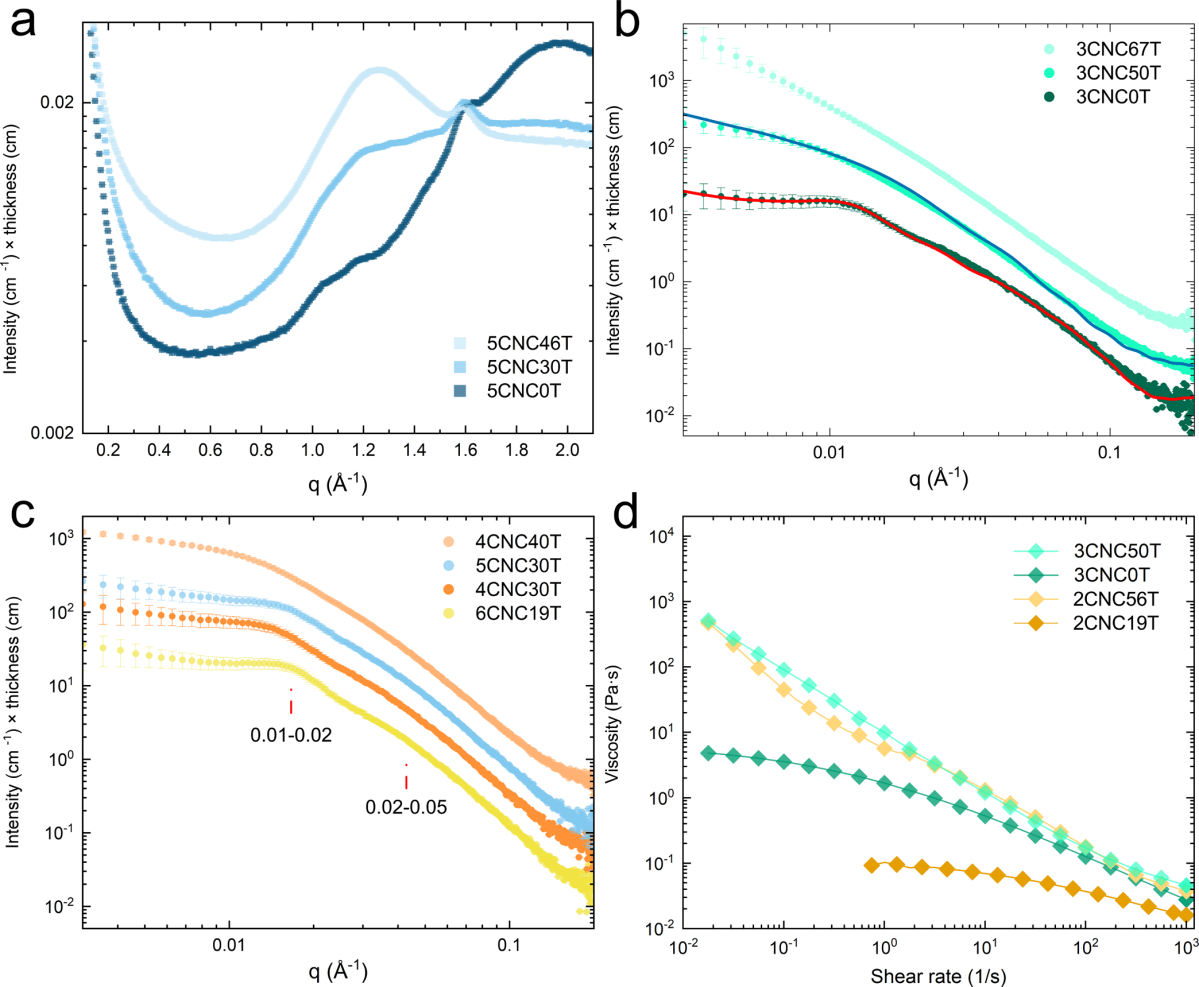
Submicron analysis of suspensions using (a) WAXS diffractogram of 5 wt % CNCs in water with 0, 30, and 46 wt % TBA as a codispersant. SAXS diffractograms for CNC-water-TBA suspensions of (b) 3 wt % CNCs with 0, 50, and 67 wt % TBA and (c) TBA additions of 19–40 wt %. The lines shown in (b) represent fits to a parallelepiped form factor with (red) and without (blue) hard-sphere structure factor. The red lines in (c) point to the broad peaks representative of CNC packing distance. The 1D SAXS intensities were shifted vertically for clarity. (d) Shear viscosity measurements with TBA concentration of 0–50 wt %. The naming convention is given as *x*CNCyT, where *x* is the CNC concentration in wt %, and *y* is the TBA concentration in wt %.

WAXS did not reveal interactions between TBA and CNC surfaces; therefore, Quartz Crystal Microbalance with Dissipation monitoring (QCM-D) and Surface Plasmon Resonance (SPR) analysis were performed using butanol and propanol isomers. The octanol–water partition coefficient of TBA (*K*_OW_) is analogous to 1-propanol at 0.43, in contrast to the higher coefficient displayed by 2-butanol at 0.75, and a lower coefficient by 2-propanol at 0.12.^34^ Despite exhibiting lower hydrophobicity relative to 2-butanol, TBA demonstrated comparable levels of absorption within the CNC film (Supporting Information: Comparison of solvents in QCM-D, Figure S3 and SPR, Figure S4). This observation suggests a strong attractive interaction, such as the adsorption of TBA on the CNC surface due to the tertiary spatial conformation and lack of micelle formation capacity, rather than hydrophobicity alone.^35,36^

The 3CNC0T suspension exhibited a broad peak at 0.01–0.02 Å^–1^ and a shoulder at 0.02–0.05 Å^–1^ in the 1D SAXS intensity pattern, which became more pronounced when displayed in Lorentz corrected plots (Figure 1b and Supporting Information: SAXS of 0 wt % TBA with CNCs, Figure S5). The broad peak at 0.01–0.02 Å^–1^ is taken to represent the CNC packing distance,^14,37^ whereas the 2:1 *q*-ratio indicates that the shoulder at 0.02–0.05 Å^–1^ corresponds to a second-order scattering peak derived from the same, presumably lamellar periodic structure.^37,38^

Based on this interpretation and following the approach of Munier et al.,^39^ the SAXS intensities originating from the CNCs in water with no TBA were fitted using a parallelepiped form factor and hard-sphere structure factor. The length was fixed at 183 nm, taken from atomic force microscopy (AFM) analysis.^40^ The fitting results of the most dilute CNC water suspension containing 3 wt % CNCs (3CNC0T) correspond to parallelepipeds with thickness of 3.8 ± 1.3 nm and width of 20.1 ± 1.7 nm, packed with an effective radius of 24.6 ± 0.7 nm (Figure 1b). AFM measurements confirmed the expected CNC diameter of 4 ± 1 nm.^41^ The fitted parallelepiped width therefore indicates that CNCs are packed with a lateral width consisting of several CNCs.^42,43^

2D SAXS intensity patterns (Supporting Information: 2D SAXS of all compositions, Figure S6) revealed anisotropy at 5 wt % CNC and above, indicating an alignment of structures in domains at the nanoscale (averaged over the size of the beam, 0.2 mm).^37^ Intensity vs azimuthal angle data indicated that anisotropy progressively intensifies beyond the 5 wt % CNC threshold. The shift of both peaks toward higher *q* was interpreted as decreased CNC distances with increasing concentration.^14^ Circular averaging of the data according to De France et al. corroborated this observation, where anisotropy became apparent above >3 wt % CNC content and CNCs were subsequently disturbed upon addition of >30 wt % TBA (Supporting Information Table S1).^44^ Hence, in the absence of TBA, CNC structures in water exhibited denser packing and increased anisotropy with higher CNC concentrations, aligning with findings reported in the literature.^37,39^

The addition of up to 30 wt % TBA caused negligible changes to the SAXS intensity curve compared to CNC-water suspensions without TBA (3CNC0T in Figure 1b vs 4CNC30T in Figure 1c). The two broad peaks at 0.01–0.02 and 0.02–0.05 Å^–1^ were still pronounced, indicating that the packing of the CNCs remained rather unchanged. Moreover, 2D scattering patterns in the 0–30 wt % TBA range at CNC concentrations at 5% and above displayed anisotropic alignment (Supporting Information: 2D SAXS of all compositions, Figure S6). At 40 wt % TBA and above, the two peaks decreased in intensity and no anisotropy could be observed (Figure 1b,c). This indicates a disappearance of structural alignment and less ordered packing as inter-CNC distances are no longer fixed, causing the system to act as a percolated network. Notably, 4CNC30T was anisotropic while 4CNC40T displayed nearly isotropic behavior.

At 50 wt % TBA, the broad CNC alignment peaks disappeared (Figure 1b) and a low *q* (<0.009 Å^–1^) intensity decay of *q* ^–1^ was obtained (Figure 1b). This indicates that CNCs exist isotropically in the suspension and show no periodic structuring. The 3CNC50T suspension could be modeled as polydisperse parallelepipeds without a hard-sphere structure factor, hence no effective radius could be obtained. The fitting corresponds to parallelepipeds with a thickness of 3.2 ± 0.3 nm, log-normally distributed with a polydispersity 0.55, and width of 26.8 ± 3.7 nm. The length was fixed again at 183 nm. Increasing the concentration of TBA to 67 wt % in 3CNC67T resulted in a similarly featureless curve as 50 wt % at high and medium *q*, but with a steeper *q* ^–2.2^ decay at low *q* (Figure 1b). This increase in slope could indicate the presence of aggregated clusters of smaller structural subunits.

The power law fits of the low *q* decay, *I*(*q*) ∝ *q* ^–α^, within the range of 0.0041 to 0.01 Å^–1^ indicated a constant *q* decay between *q* ^–0.2^ and *q* ^–0.6^ at TBA concentrations below 40 wt % (Figure 2a). This behavior transitioned to a *q* decay close to *q* ^–1^ at 50 wt % TBA, with even more pronounced decay at higher TBA concentrations. It suggests that, regardless of CNC concentration, the CNC network transitioned from ordered packing to percolated structures and subsequently to larger aggregated clusters with increasing TBA concentration.

**Figure 2:**
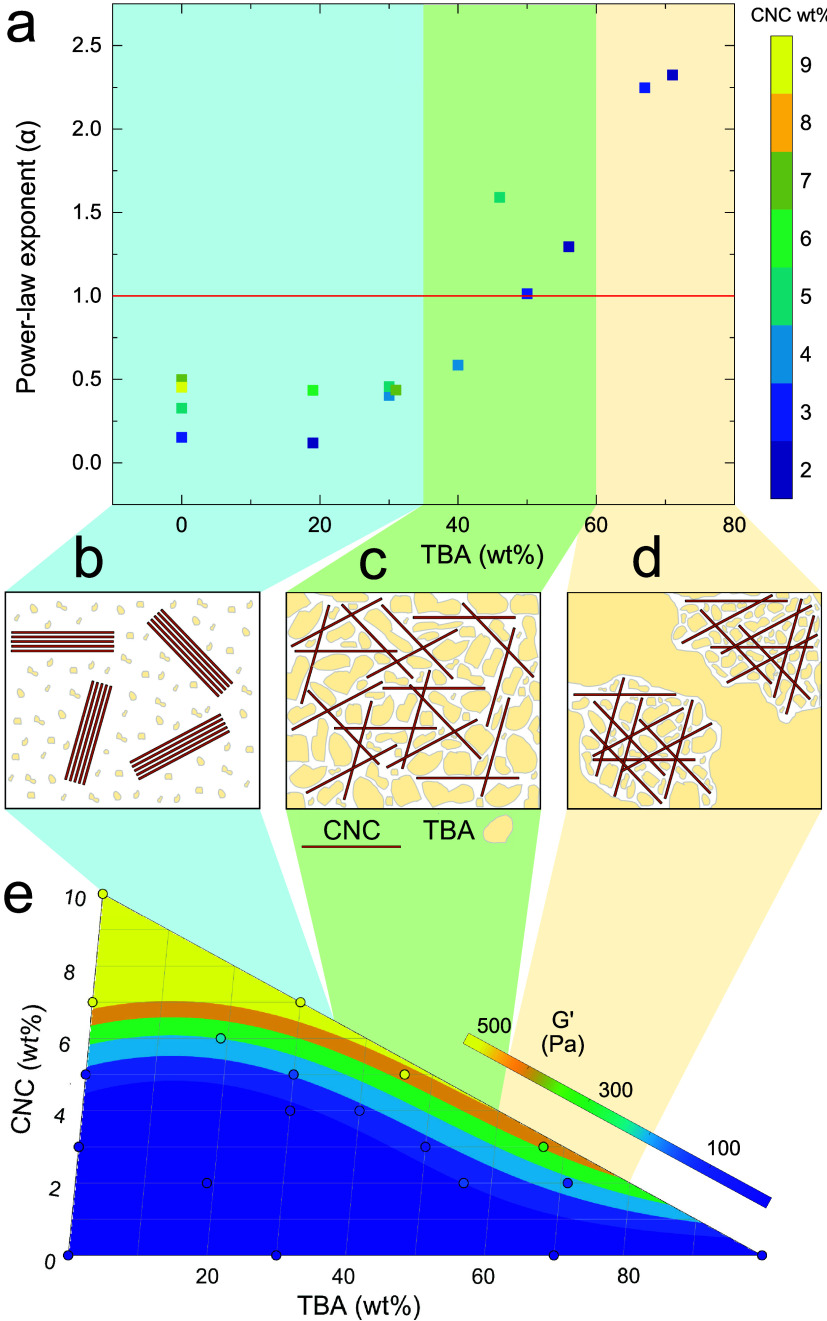
Combination chart of (a) the power-law exponent, α, as a function of TBA content. The red line denotes the transition from ordered CNC packing (α ≤ 1) to increasing degrees of aggregation. The blue and green regions depict the concentrations of TBA corresponding to these structuring modes. (b–d) Schematic representation of CNC assembly. (e) Ternary contour plot of *G*′ (Pa) in the linear region during strain sweep measurements. The contour areas were obtained in JMP by a fitted model, with yellow corresponding to higher values and blue to lower values.

#### Rheological Behavior of CNC Suspensions

Water, TBA, and their blends (excluding CNCs) expressed Newtonian rheological behavior. In agreement with values reported by Furukawa et al.,^45^ shear viscosities increased to a maximum at 70 wt % addition of TBA (Supporting Information: Shear viscosity plots, Figure S7). At low CNC (<5 wt %) and TBA (<30 wt %) concentrations, suspensions displayed isotropic shear behavior characterized by a Newtonian plateau at low shear rates followed by shear thinning at higher rates (Figure 1d). However, with the addition of up to 60 wt % TBA, consistent shear-thinning behavior was observed at all shear rates. The presence of TBA thereby intensifies interparticle interactions.

Across all CNC concentrations, TBA concentrations up to 60 wt % corresponded with a significant rise in viscosity and storage modulus (*G*′), while tan δ decreased, indicating a transition to more elastic behavior (Table 1 and Figure 2e). The presence of a crossover point for the curves of *G*′ and loss modulus (*G*″) curves suggests an improved flexibility and resilience of the network structure. The increase in structural integrity was analogous to that achieved by augmenting the concentration of CNCs (Figure 2e and Supporting Information: Strain sweep plots, Figure S8). Beyond 60 wt % TBA, phase separation was visually observed, and the strain at *G*′ = *G*″ decreased indicating a higher susceptibility to breakage at lower strains.

**Table 1:** Selected Rheological Parameters from Linear Viscoelastic Strain Sweeps of Suspensions Containing 2 and 3 wt % CNC: Viscosity at 1 s^–1^, *G*′, tan δ, and Strain at *G*″ = *G*′^a^

CNC (wt %)	TBA (wt %)	viscosity (Pa·s)	*G*′ (Pa)	tan δ	strain (%)
2	19	0.1	0.3	1.71	
2	56	5.7	139	0.20	43
2	71	0.3	*	*	15
3	0	1.7	4	1.15	
3	50	9.9	66	0.13	93
3	67	1.2	*	*	16
5	0	12.2	40	0.63	21
5	30	19.6	141	0.23	54
5	46	57.3	989	0.11	117
7	0	71.2	581	0.12	67

a Values that could not be determined reliably from phase separated suspensions are noted with a *.

To elucidate this phenomenon, all suspensions were fitted for their rheological responses using JMP and depicted in ternary diagrams (Figure 2e and Supporting Information: Ternary plot of viscosities, Figure S9). Figure 2e illustrates the ternary diagram of *G*′ derived from the viscoelastic linear region of the strain sweep curve. The model describes a positive linear interaction between CNCs and TBA having a high significance toward the response (*p*-value = 0.02). Moreover, interactions between CNCs had a quadratic effect on the behavior. Individual effects were not significant, indicating that their influence on the response is primarily mediated through interactions rather than individual concentrations.

#### Morphology and Properties of CNC Solid Foams

Solid foams prepared from CNC-water suspensions formed macroscopic lamellar morphologies observable by SEM (Figure 3a and Supporting Information: SEM images of 0 wt % TBA with CNCs, Figure S10). The CNCs aggregated into sheets, forming a poorly interconnected network throughout the bulk of the solid foam. With the addition of <30 wt % concentration of TBA, the lamellar sheets were partially disrupted, and submicron pores were observed throughout the network (Figure 3b). Within the TBA concentration range of 30–50 wt %, needle-shaped structures emerged, characterized by a homogeneously distributed rough texture (Figure 3c). At TBA concentrations exceeding 60 wt %, larger aggregated macro-structures developed with microporous inclusions and dispersed moss-like external features (Figure 3d). The configuration of the molds (thick walls and thin top/bottom plates) lead to the formation of anisotropic foams due to the directional growth of ice crystals. The SEM images in Figure 3c are the most representative of the true axial view of the foams.

**Figure 3:**
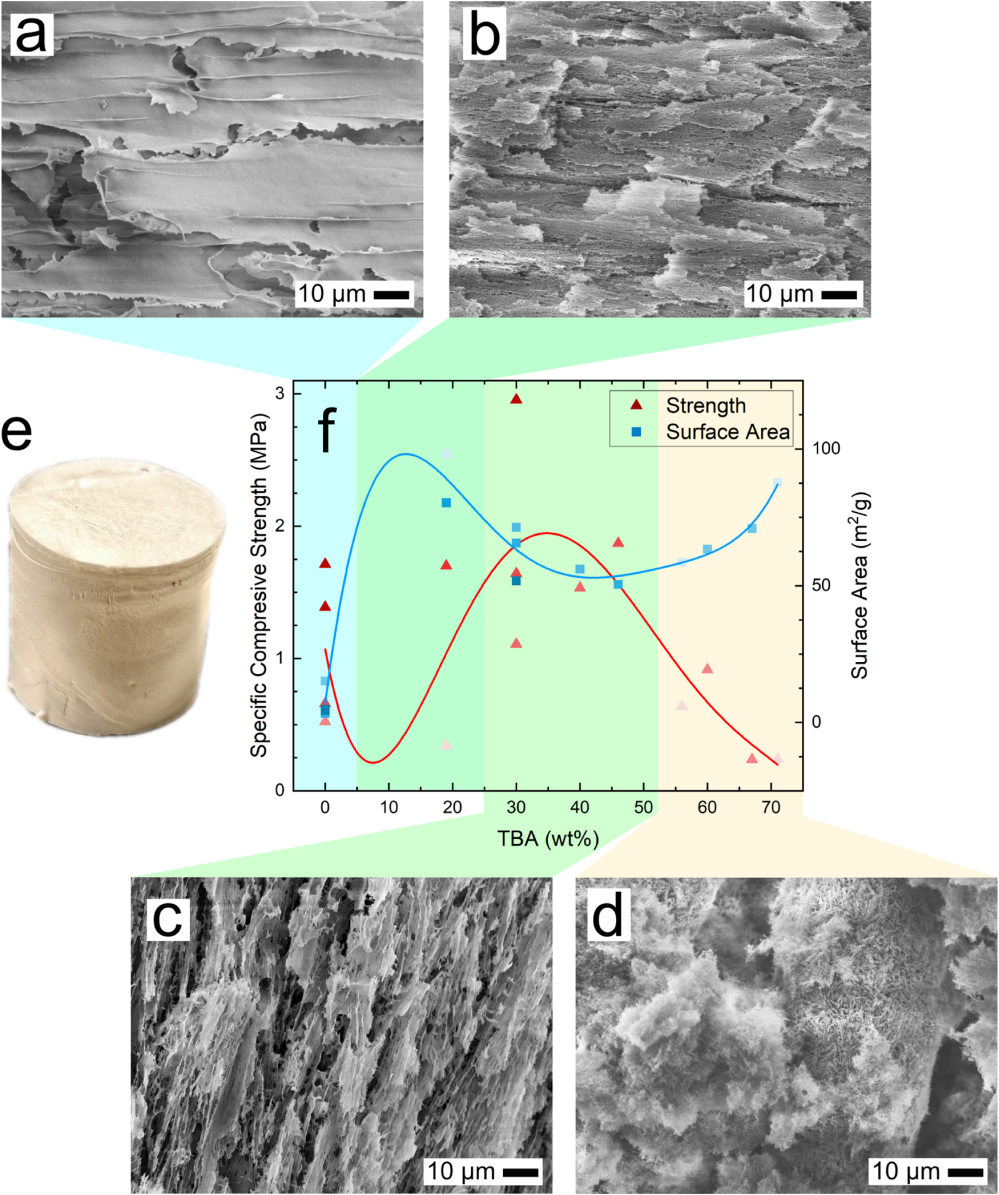
Morphologies of CNC solid foams observed via SEM at 1000× magnification for (a) 0 wt % TBA (lamellar) rotated 90°, (b) < 30 wt % TBA (porous lamellar) rotated 90°, (c) 30–50 wt % TBA (needle-like) rotated 15°, and (d) > 60 wt % TBA (aggregated) rotated 15°. The freeze-dried solid foam for 7CNC30T is shown in (e) and the correlation between surface area and specific compressive strength at 10% strain with respect to TBA wt % is presented in (f). The trend lines are meant as guides for the eye rather than true fits. Varying CNC concentrations are indicated by the brightness of the data points, where darker points represent a higher wt % of CNCs.

Increasing CNC concentration in solid foams made without TBA led to augmented compressive properties as CNC–CNC contacts reinforced the sheet structure (Supporting Information: All foam properties, Table S2). The increased CNC content simultaneously reduced porosity and surface area, particularly for foams containing more than 5 wt % CNCs (<5 m^2^/g). The introduction of TBA resulted in solid foams with increased surface area. The addition of <30 wt % TBA induced negligible changes in compressive properties while surface area reached a local maxima (Figure 3b and Supporting Information: Ternary plots of surface area and porosity, Figure S11). Surface areas peaked at low (20 wt %) and high (>60 wt %) concentrations of TBA.

While increasing the concentration of CNCs improved the compressive strength of the solid foams, the incorporation of TBA could achieve comparable or superior strength. For instance, foams obtained from 7 and 10 wt % of CNCs in water (7CNC0T, 10CNC0T) exhibited specific moduli of 22 and 40 MPa, respectively, whereas 4 wt % CNC with 40 wt % of TBA (4CNC40T) presented a specific modulus of 36 MPa (Supporting Information: All foam properties, Table S2 and ternary plots of modulus, strength and energy absorption, Figure S12). Increasing TBA concentration by up to 50 wt % consistently compensated for the mechanical deficit associated with lower CNC content. This observation was paired with the systematic morphological transition from lamellar sheets to needle-like structures. Addition of 30 wt % TBA to 7CNC0T resulted in significant improvements, marked by a 180% increase in strength and stiffness, a 120% increase in energy absorption, and a 10-fold increase in surface area (from 5 to 52 m^2^/g) (Figure 3c,3f, Supporting Information: All foam properties, Table S2 and ternary plots, Figures S11 and S12).

The integration of TBA as a codispersant demonstrated an inverse relationship between surface area and mechanical properties, where an increase in surface area corresponded with a decrease in mechanical properties, and vice versa. The surface area and specific compressive strength showed cubic and quadratic correlations to TBA concentration, respectively (Figure 3f). The surface area reached its apex at 20 and 70 wt % TBA content, while mechanical properties remained similar to the solid foams with 0 wt % TBA. Incorporation of 30–50 wt % TBA in CNC suspensions resulted in outstanding compressive mechanical performance. Solid foams in this range can be tailored toward applications demanding high specific mechanical performance while preserving a greater surface area than foams devoid of TBA.

### Mechanisms of Structure Formation

#### CNC-TBA Suspensions

X-ray scattering and rheology depict the intricate interactions between CNCs, TBA, and water at submicron to macroscopic length scales. In water, CNCs are organized into anisotropic structures that can be probed with X-ray scattering (Figure 2a). As CNC concentration increases, the packing density and order of these structures become more pronounced.

The packing, anisotropy and the rheological properties of CNC networks are unaffected with up to 30 wt % addition of TBA (Figure 1, 2b). Above 40 wt % TBA, the ordered arrangement of >3 wt % CNCs becomes disrupted (Figure 1b,c) as evidenced by isotropic 2D scattering patterns, low *q* decay slope (*q* ^–1^) and increased suspension network strength (Figure 2c). Consequently, both viscosity and *G*′ increased, indicative of augmented network strength, attributed to (i) interparticle interactions and (ii) the formation of a percolated CNC network. Therefore, the altered liquid-phase structuring suggests that TBA disrupts the ordered packing of CNCs to form a randomly dispersed, strengthened interconnected network which enhances the structural integrity and stability of the suspension.^3,4^ These phenomena are attributed to the following mechanisms:

i) TBA forms liquid structures with free water, proportionally decreasing the relative permittivity of the medium with the addition of TBA.^46,47^ The lower polarizability leads to a strengthened screening of the electric potential between charged CNCs, reducing the Debye length. The reduced Debye length allows CNC to be closer to each other, increasing the likelihood of hydrogen bonding between them and forming stronger networks.

ii) According to the literature and corroborated by our SPR and QCM-D analysis, the tertiary hydrophobic volume of TBA adsorbs onto hydrophobic CNC interfaces, resulting in an increase in CNC hydrophilicity due to the orientation of hydroxyl groups toward the bulk water phase.^48,49^ The adsorption is both entropy and enthalpy driven, as water molecules are liberated from surfaces in favor of hydrophobic CNC-TBA interactions, leading to a spontaneous thermodynamic process.^50–52^ Therefore, attractive interactions from London and van der Waals forces along with long-range electrostatic repulsion are suppressed, allowing CNCs to disperse isotropically into percolated networks.^53^

At concentrations above 60 wt %, TBA displays a dominant volumetric presence within the suspension. CNCs and water become enveloped by TBA-rich regions, leading to the depletion flocculation of CNCs into disparate clusters (Figure 2d).^54^ TBA competes with bound water at hydrophilic CNC surfaces, and further reduction in Debye length leads to diminished repulsion between CNCs, causing destabilization and aggregation in the dispersed isotropic network as suggested by the observation of low *q* decay exceeding *q* ^–2^ (Figure 2a). This ultimately causes the suspensions to undergo phase separation when left to rest.

#### Freeze-Dried CNC Foams

The decisive step influencing the morphology of the freeze-dried suspensions resides in the freezing process. During freezing, the growth of ice crystals pushes CNC structures present in the fluid state into the interstitial regions between the crystals. The result of this process is observable as the diverse morphologies observed via SEM imaging of the solid foams (Figure 3a–d). It is well documented that the addition of or solvent exchange with TBA leads to freeze-dried CNC foams with increased surface area.^31,55,56^ TBA and water freeze at their corresponding freezing points unless the mixture reaches its eutectic points at approximately 20 and 90 wt % TBA.^5,29,30,57^ It is suggested that the eutectic mixture between TBA and water creates hydrogen-bonded liquid microclusters, which limits the growth of ice crystals.^30,57^ This phenomenon was observed for foams containing 19 wt % TBA (Figure 3b). Microporous features could be seen at 19 wt % TBA compared to the relatively smooth lamellar sheets at 0 wt % TBA (Figure 3a), which are ascribed to the freezing of the TBA-water microclusters. These micropores led to an increase in surface area while preserving the macroscopic lamellar structuring. Borisova et al.^29^ reported findings supporting this hypothesis, wherein the addition of 30 and 90 wt % TBA generated the smallest pore diameters in polysaccharide aerogels.

Suspensions at 30–50 wt % TBA are between the eutectic points, causing TBA to freeze first and form dendritic crystals.^57^ The needle-shaped pores, which appear in the morphology of solid foams after freeze-drying, support the conclusion that the solid foam structure is guided by the formation of crystals in the liquid-phase (Figure 3c). Within this concentration range, the liquid-phase structuring of TBA and water has been shown to be a bicontinuous microemulsion.^3^ Therefore, suspensions were expected to maintain the liquid-phase structuring when frozen rapidly using liquid nitrogen due to simultaneous prolific nucleation.^58^ However, this assumption was challenged by the presence of an external mold containing the suspensions, which induced temperature gradients. This is supported by observations from Cai et al.^59^ who noted an increase in pore diameter toward the center of a freeze-dried structure, attributable to slower growth of ice crystals. Thus, the macroscopic anisotropy observed in Figure 3c is ascribed to directional crystal growth from temperature gradients, particularly in the axial cut through the center of the foams where the images were taken. The anisotropy of both pore direction and aspect ratio provides the foams drastic improvements in the compressive strength parallel to the direction of loading. Anisotropic ice-templated foams studied by both Darpentigny et al. (2020)^56^ and Ruiz-Caldas et al. (2024)^60^ had a similar morphology to those shown here. Darpentigny et al. notably used tunicated CNCs, which naturally formed a ”netted” structure similar to our foams with 30–50 wt % TBA added. The increase in strength over cryogels from cotton CNCs was ascribed to the change in structure as well as the significant increase in crystallinity. The CNC cryogels produced by Ruiz-Caldas et al. (3 and 5 wt % CNCs) without TBA had remarkably similar stress–strain curves to those produced here, giving credence to the strengthening effect of TBA.

The mechanical performance and surface area of solid foams can be understood by merging the concepts of the liquid-phase and freeze-induced structuring. The increased surface area is due to the TBA-water interactions disrupting the long-range ice crystal formation during the freezing process. Surface area lowered from 20% to 50% as the TBA-water microclusters became larger bicontinuous regions, increasing crystal size. Compressive strength likely increased as CNCs became isotropically dispersed within pore walls (>30 wt % TBA). The isotropic arrangement would increase intercrystal connectivity, however minor changes to the macro-structure in this range may be the true determinant force and cannot be discounted. Given that surface area lowered by 15 m^2^/g when comparing 4CNC30T versus 4CNC40T, the CNCs may have packed more densely due to the size of the crystal formation. This densification leads to reinforced pore walls, as a result of increased capability for stress distribution (Supporting Information: All foam properties, Table S2).^61,62^ Therefore, it is unclear the true effect of liquid-state CNC structuring on the final properties of the solid foams.

Increasing the TBA concentration to 70 wt %, led to the formation of large disconnected structures of isotropically dispersed CNCs with high porosity due to TBA-water microclusters (Figure 3d). This foam network fails to act as a unified macro-structure from a lack of poor long-range structural support and yet maintains a high surface area as a result of the freezing behavior.

## Conclusions

This study demonstrates the role of TBA as a cosolvent in modulating the intricate nano- to macro- structural properties of CNC suspensions and the resulting freeze-dried foams, consequently enhancing their robustness and functionality. Addition of 20 wt % TBA does not hinder the ordered structuring of CNC suspensions as evidenced by small-angle X-ray scattering and rheology characterization. Nevertheless, during freezing, the mixture’s eutectic nature causes microporous inclusions to form, significantly augmenting surface area within the resulting solid foams. Elevating TBA concentrations above 30 wt % triggers a disruption of the assembly of CNCs, driven by the adsorption of TBA to hydrophobic CNC surfaces. This allows the CNCs to escape constrained periodic structuring. Moreover, the Debye length of the medium diminishes, reducing distances between CNCs and forming reinforced isotropic networks. The distinct liquid-phase organization and noneutectic nature of TBA-water suspensions direct the crystal growth. This leads to the formation of needle-shaped pores and highly interconnected CNC pore walls in freeze-dried solid foams with addition of 30–50 wt % TBA. Consequently, the compressive strength of the foams peaks in this TBA concentration range, and surface area becomes considerably larger than foams made without TBA. When TBA exceeds 60 wt %, the stability of the suspension becomes compromised as TBA induces the formation of disparate regions containing aggregated CNCs which easily phase separate. This destabilized network structure freeze-dries into porous isotropic structures displaying high porosity and poor compressive strength. Alcohols present an exceptionally effective method to organize amphiphilic cellulose particles. The structure and properties of suspensions and freeze-dried foams can be tailored using TBA as a structuring agent. This study paves the way to using solvent-mediated design strategies in the synthesis and applications of nanomaterials.

## Supplementary Material



## Notes

Includes ternary plots describing CNC-TBA-water compositions and viscosities, and freeze-dried foam surface area, porosity, compressive modulus, strength and energy absorption analysis. QCM-D and SPR of the suspensions are included, as well as the SAXS and WAXS diffractograms including 2D SAXS intensity patterns and tabulated circular averaging values. Measurements of rheology and viscosity of the strain sweep values, and SEM images of foam morphologies without TBA are included (PDF)
