# Supplementary material for: Tert-Butanol as a Structuring Agent for Cellulose Nanocrystal Fluids and Foams
Source: Biomacromolecules. 2025 Jul 18;26(9):5591–600. doi: 10.1021/acs.biomac.5c00184 (PMC12421691; doi:10.1021/acs.biomac.5c00184)
Supplement: Supplementary file 1 [file bm5c00184_si_001.pdf]

Supporting Information for

Tert-butanol as a structuring agent for cellulose nanocrystal fluids and foams

Saul Llàcer Navarro[a,b], Eliott Orzan[a], Ratchawit Janewithayapun[a], Paavo Penttilä[c], John Andersson[a], Anna Ström[a], Roland Kádár[d], and Tiina Nypelö[a,b,c*]

[a]*Department of Chemistry and Chemical Engineering, Chalmers University of Technology, Kemivägen, 41296 Gothenburg, Sweden*

[b]*Wallenberg Wood Science Center (WWSC), Chalmers University of Technology, 41296 Gothenburg, Sweden*

[c]*Department of Bioproducts and Biosystems, Aalto University, Vuorimiehentie 1, 02150 Espoo, Finland*

[d]*Department of Industrial Material Science, Chalmers University of Technology, Kemivägen, 41296 Gothenburg, Sweden*

*Corresponding author email: tiina.nypelo@aalto.fi

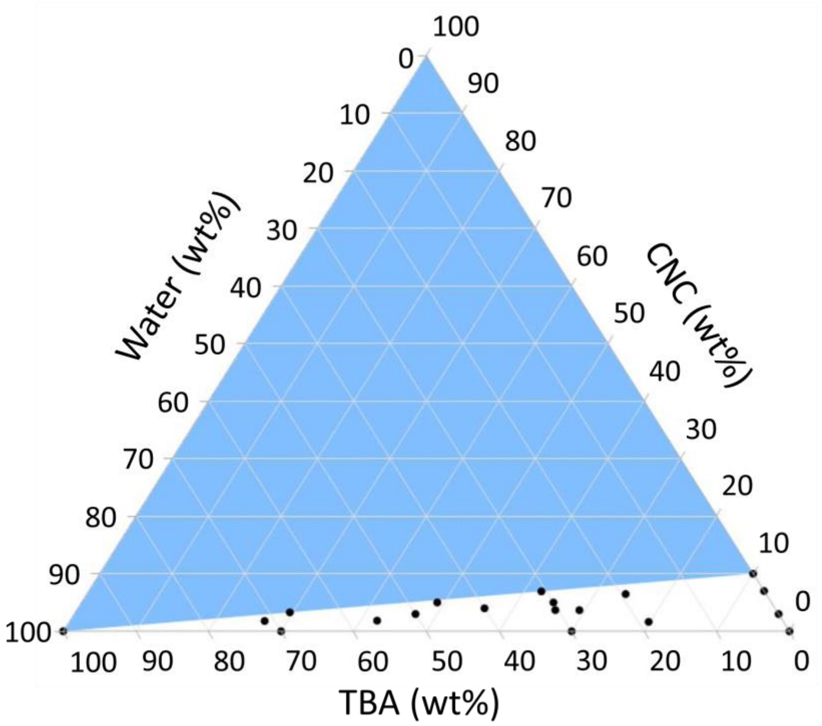

Figure S1. Ternary plot summarizing the suspensions containing selected concentrations of CNCs, water, and TBA assessed in this study (black dots). The white region represents the experimental range accessible within the limitations of the stock suspensions. The naming convention is given as *x*CNC*y*T, where *x* is the CNC concentration in wt%, and *y* is the TBA concentration in wt%.

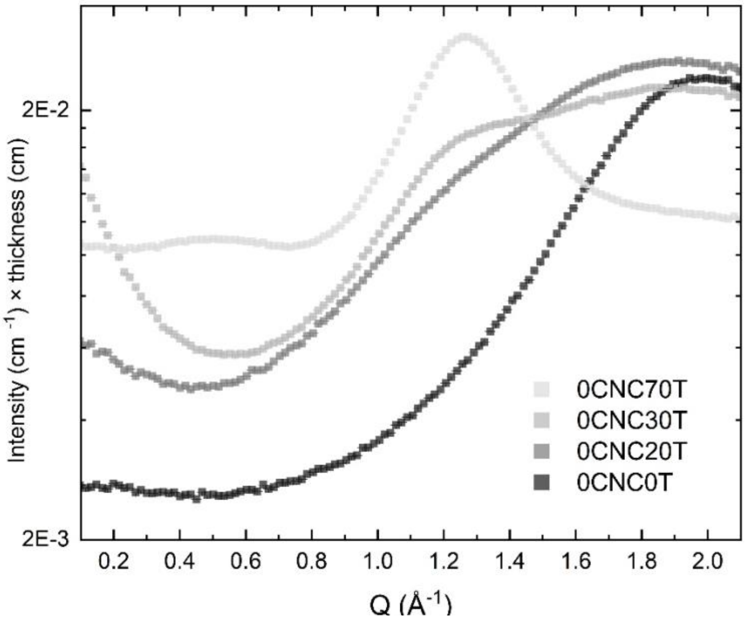

Figure S2. WAXS diffractogram of water with 0, 20, 30, and 70 wt% TBA as a co-dispersant. The peak at 2 $Å^{-1}$ corresponds to O-O distances in water, while subsequent peaks at 1.25 and 0.5 $Å^{-1}$ originate from hydrophobic interactions between aliphatic TBA groups and hydrophilic interactions between TBA hydroxyl groups, respectively (Buchecker et al., 2017). The appearance of an upturn below 0.5 $Å^{-1}$ when CNCs are present corresponds to the start of mesostructure scattering in the SAXS range (5CNC30T and 0CNC30T).

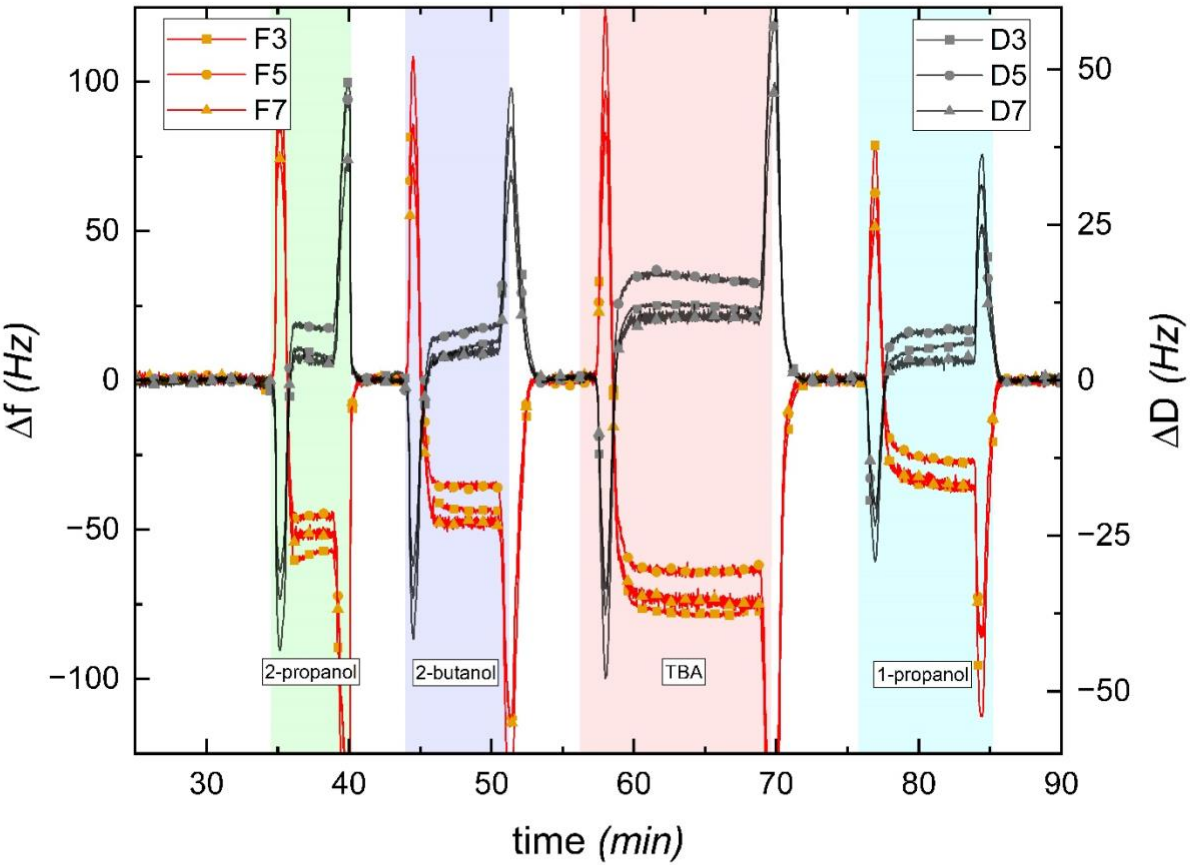

Figure S3. Baseline-corrected frequency (Δf, left axis) and dissipation (ΔD, right axis) shifts for various butanol and propanol isomers, measured using QCM-D on a CNC spin-coated silicon oxide sensor. Data are shown as the net difference from an uncoated reference sensor to qualitatively isolate the effects of solvent adsorption, swelling, and structural reorganization of the CNC layer. Baseline correction was performed using MQ water injections, with a spline fit applied during these phases to ensure consistency. The frequency shifts (Δf, orange) and dissipation shifts (ΔD, gray/black) for different harmonic overtones (F3, F5, F7 and D3, D5, D7, respectively) were monitored during sequential injections of 2-propanol, 2-butanol, TBA, and 1-propanol (12 wt%). A frequency decrease (Δf) represents solvent uptake or mass adsorption by the CNC layer, while shifts in dissipation (ΔD) reflect changes in the viscoelasticity of the layer, with negative and positive shifts corresponding to a more rigid film or a softer film, respectively. Compared to the other tested solvent compositions, the larger frequency decrease and larger dissipation increase was observed for TBA, suggesting stronger interactions with the CNC layer, likely driven by TBA sterically matching both the hydrophobic regions and hydrogen-bonding donor and acceptor groups of the CNC. This adsorption also disrupts the interfacial interactions between CNC’s, which together with the increased solvent uptake causes the layer to soften. These results support our hypothesis that the interactions between CNC and solvents depend strongly on the solvent's polarity, hydrophobicity, and molecular structure.

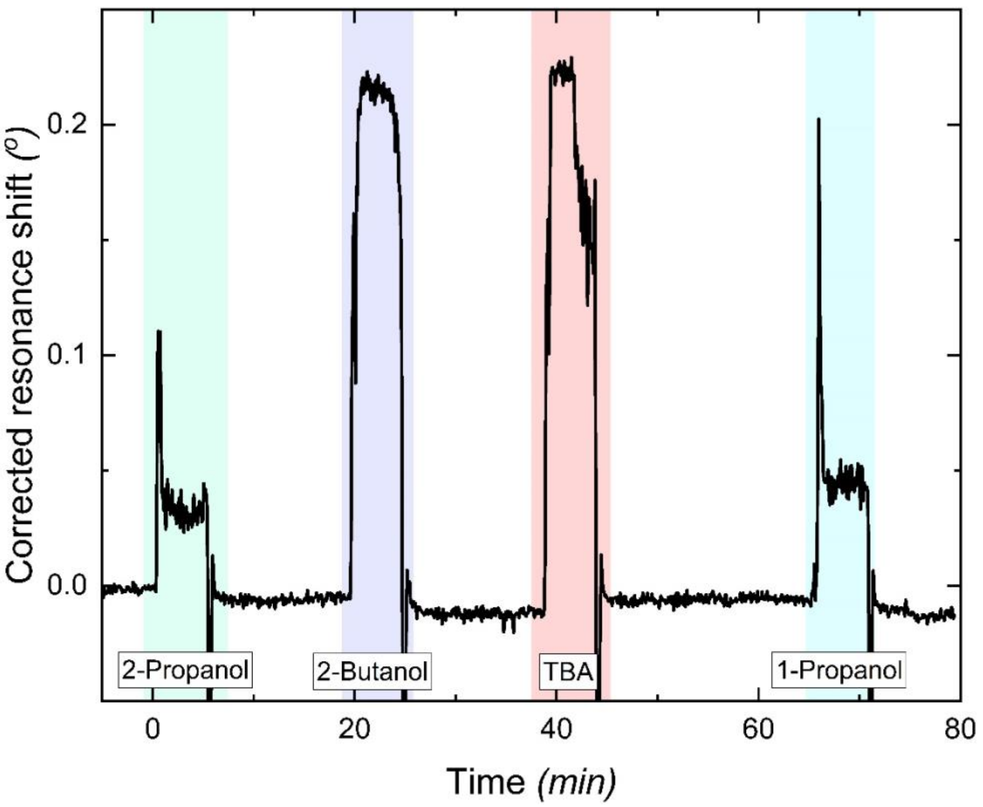

Figure S4. Bulk corrected resonance shift (Svirelis, et al., 2022) of various butanol and propanol isomers on a CNC spin coated silicon oxide sensor as measured by Surface Plasmon Resonance (SPR). An increase in the corrected resonance shift corresponds to an increase in the refractive index close to the sensor surface. The injections of 2-propanol, 2-butanol, TBA, and 1-propanol at 12 wt% were carried out in sequence (as indicated by the colored regions). (Svirelis, et al., 2022). Baseline drift was subtracted using an exponential fit. Higher shifts can be observed for 2-butanol and TBA compared to 2-propanol and 1-propanol. These shifts can be attributed either to an increased solvent component uptake inside the CNC film or to increased deswelling of the CNC film. However, the increase in dissipation observed in the QCM-D results (Figure S3) suggest we can largely attribute the positive SPR shifts to solvent uptake.

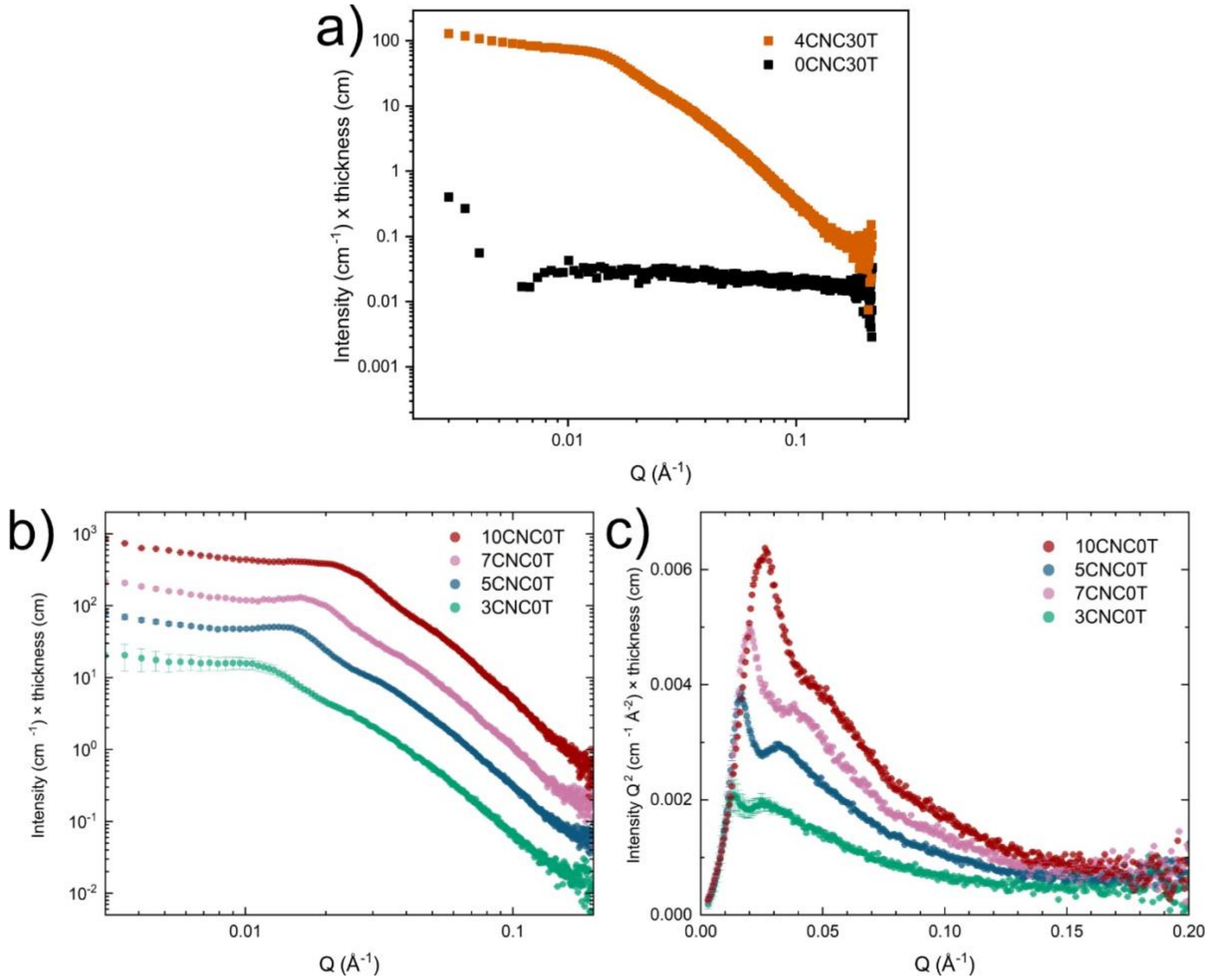

Figure S5. SAXS diffractograms. a) comparing diffraction intensities with and without CNCs. b) CNC-water suspensions at CNC concentrations (3, 5, 7, and 10 wt%). c) Lorentz corrected plot ($Q^2I(Q)$ vs. Q) for the aforementioned suspensions. Both peaks shifted to higher *q* values with increasing CNC concentration. However, the shoulder remained at a constant *q*-ratio of 2:1 to the peak. The shift of both peaks towards higher *q* was interpreted as decreased CNC distances with increasing concentration (Schütz et al., 2015).

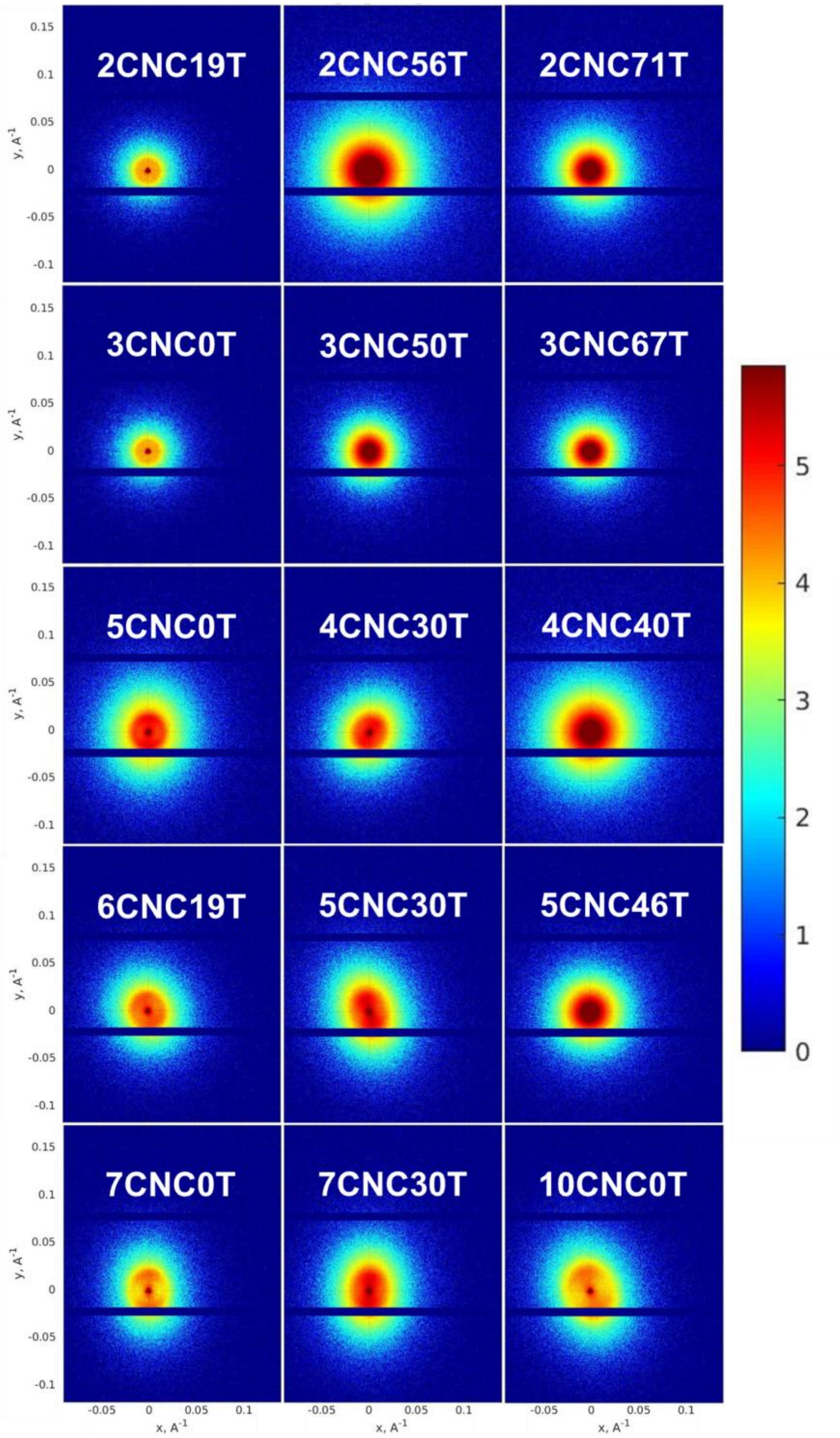

Figure S6. 2D SAXS intensity patterns for CNC-TBA-water mixtures at various CNC (2 wt% to 10 wt%) and TBA concentrations (0 wt% to 71 wt%) concentrations. The isotropic scattering at lower CNC concentrations (e.g., 2CNC19T, 3CNC0T) indicates random orientation of CNCs. With the introduction of higher CNC concentrations (e.g., 5CNC0T, 6CNC19T), anisotropy starts to appear, which is indicative of alignment or long-range ordering of CNC domains. However, at higher TBA concentrations, such as in 4CNC40T and 5CNC46T, the scattering appears more isotropic, suggesting that TBA interferes with the alignment of CNCs, preventing or disrupting the formation of ordered structures observed in CNC-water systems.

Table S1. 2D SAXS data fitting according to Equation 1 in De France et al. (2016) to yield the ad hoc order parameter (η), indicating where from 0 (isotropic) to 1 (anisotropic) the suspension is. There is a visibly significant increase starting at 4CNC30T, disappearing at 4CNC40T. The trends suggest that CNC assemble into ordered structures at > 3 wt% CNC content and are disturbed by addition of > 30 wt% TBA. The C is a scaling constant and χ is the alignment direction.

q ($Å^{-1}$)	0.01-0.02			0.02-0.05		
	**η**	C	χ	**η**	C	χ
10C0T	**0.12**	14.5	108	**0.16**	13.8	108
7C30T	**0.14**	27.0	89	**0.19**	15.9	88
7C0T	**0.14**	14.9	95	**0.20**	14.2	93
6C19T	**0.07**	20.4	111	**0.09**	12.8	114
5C46T	**0.02**	50.9	86	**0.03**	15.0	86
5C30T	**0.15**	24.6	107	**0.21**	14.2	108
5C0T	**0.09**	16.1	87	**0.10**	9.2	87
4C40T	**0.02**	26.7	80	**0.02**	9.5	73
4C30T	**0.09**	24.4	62	**0.10**	10.8	64
3C67T	**0.00**	23.9	73	**0.00**	5.3	54
3C50T	**0.03**	34.1	91	**0.03**	8.8	94
3C0T	**0.00**	15.6	117	**0.00**	5.0	75

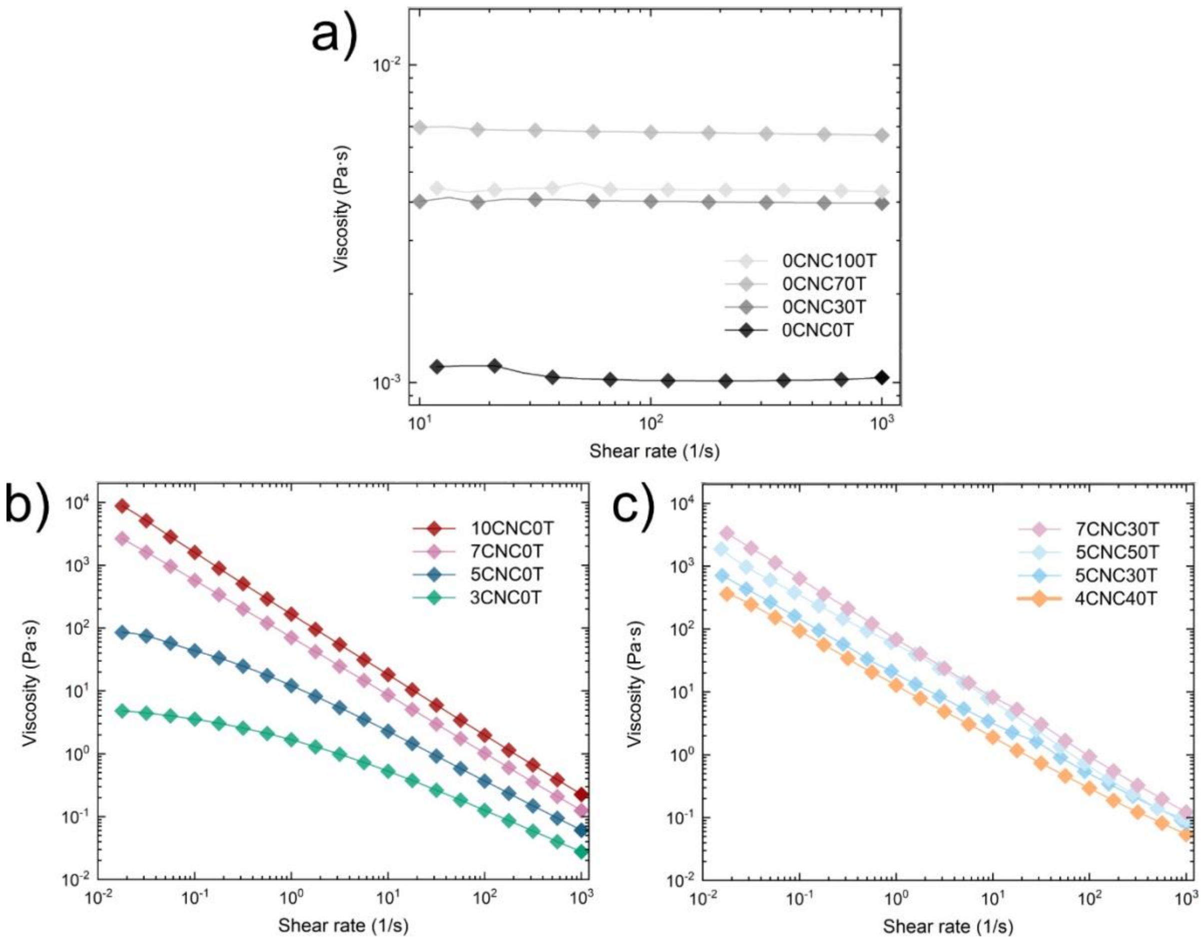

Figure S7. Viscosity measurements of CNC-TBA-water. The shear viscosity was measured at shear rates of 10-1000 s^{-1} (for solutions without CNCs) and 0.015-1000 s^{-1} (suspensions containing CNCs). (a) TBA-water systems without CNC. b) CNC-water systems with 3 to 10 wt% CNC. (c) CNC systems with 4 wt% to 7 wt% CNC and moderate TBA concentrations. Increase in TBA content was accompanied by an increase in viscosity. These suspensions showed a single shear-thinning viscosity function.

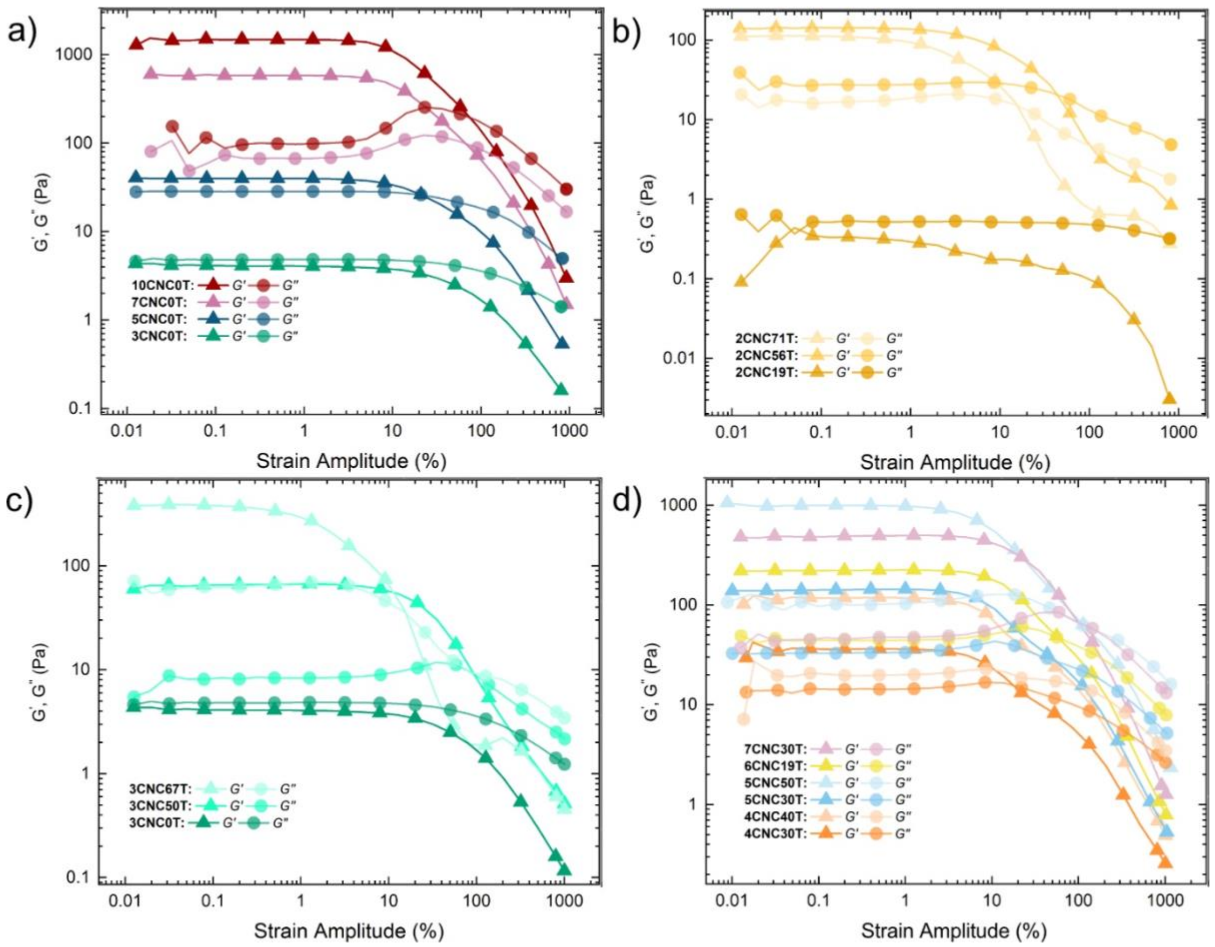

Figure S8. Shear strain sweep measurements of CNC-TBA-water suspensions. Triangles correspond to G', and circles to G''. Shear strain sweep measurements were conducted at a frequency of 1 Hz and strain of 0.01 - 800% strain. (a) CNC-water systems without TBA for CNC concentrations of 3 wt% to 10 wt% show a steady increase in G′ with increase in CNC. b) CNC systems with 2 wt% CNC and varying TBA concentrations show that TBA increases G' enhancing material's resistance to deformation. The system shows phase separation, marked by a sharp decrease in G', leading to premature yielding. (c) CNC systems with 3 wt% CNC and different TBA concentrations, also showed an increase in G' with TBA and a phase separation at high concentration (d) CNC systems with 4 wt% to 7 wt% CNC and moderate TBA concentrations. Moderate TBA concentrations (30T and 40T) reinforce the network, resulting in a higher G′ and a shift in the crossover point (G' = G'') to higher strain values. This indicates that TBA addition strengthens the CNC network up to a certain concentration, allowing the material to sustain higher deformations before transitioning to viscous behavior. Specifically, 5CNC40T demonstrates the highest G′ and the most strain-resilient structure.

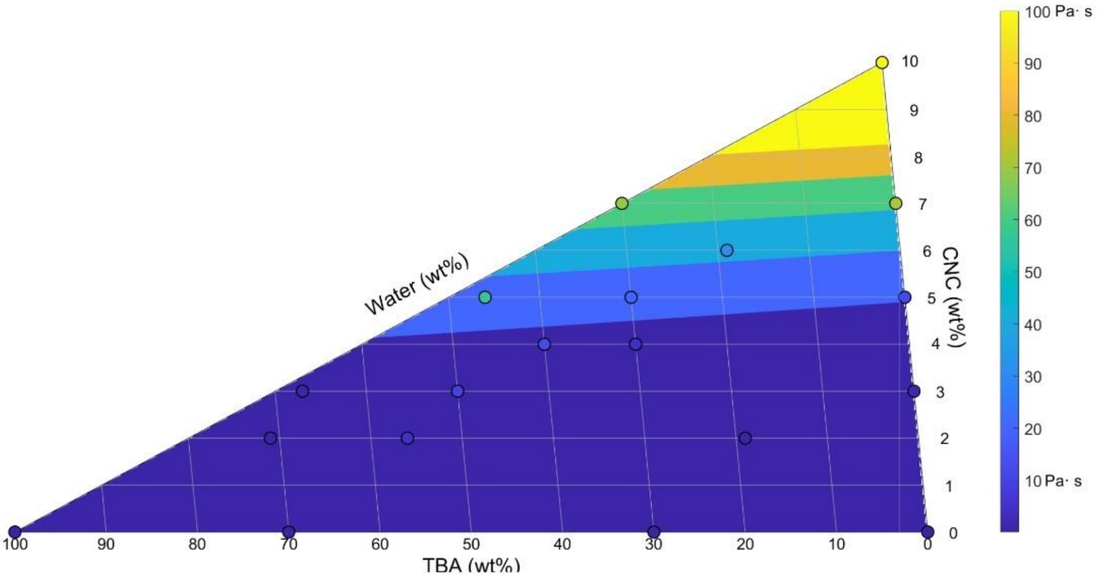

Figure S9. Ternary contour plots with selected suspensions and blends of CNC, water, and TBA. The color scale denotes the steady state shear viscosity (Pa/s) at 1 s^{-1} shear rate. The contour areas were obtained in JMP by a fitted model, with yellow corresponding to higher values and blue to lower values. The system becomes highly viscous at higher CNC concentrations and moderate water/TBA content, while lower viscosity is observed at high water or high TBA concentrations, particularly at lower CNC content.

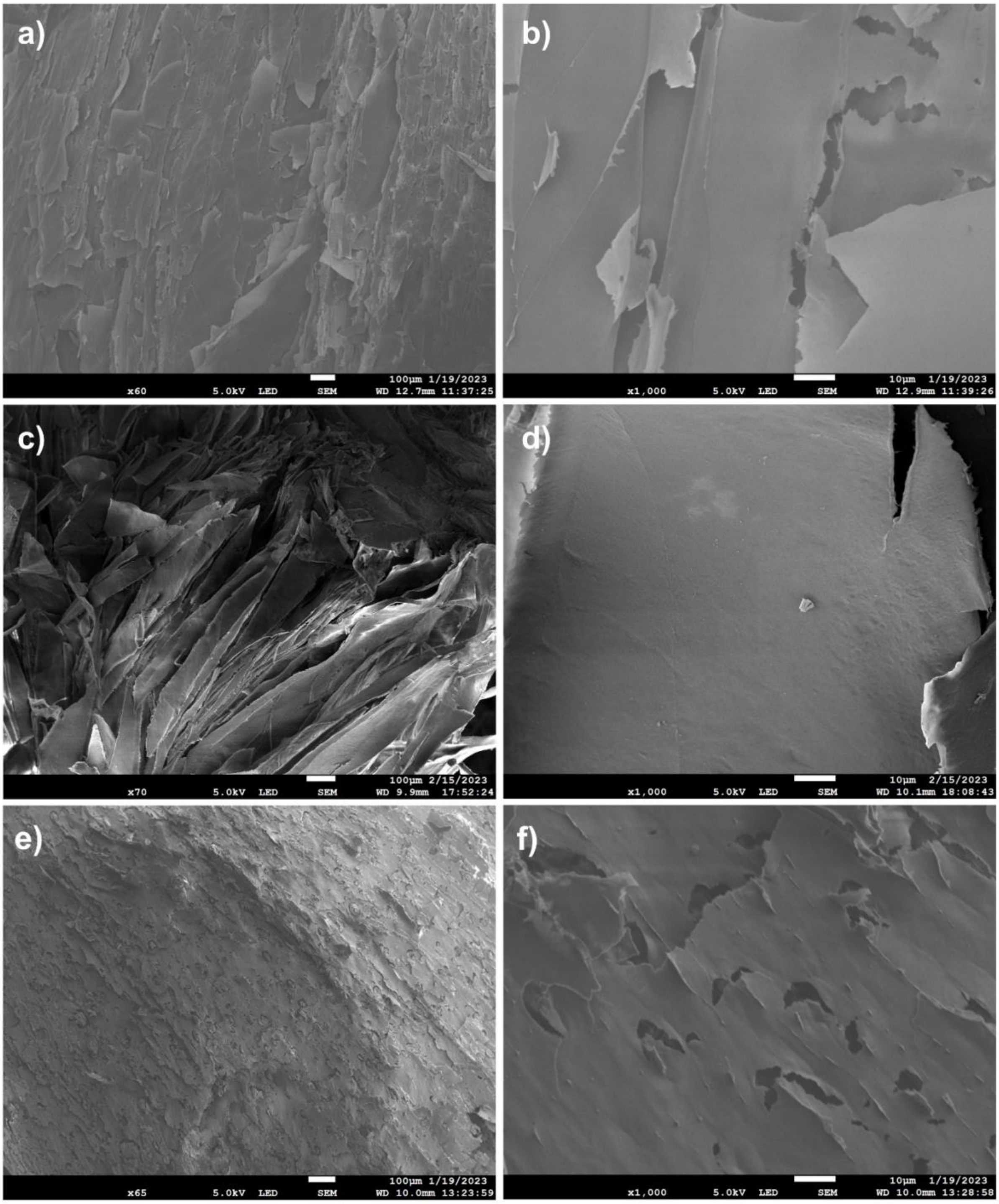

Figure S10. SEM images of solid foams made without the addition of TBA: a) 3CNC0T, 60x magnification, b) 3CNC0T, 1000x magnification, c) 5CNC0T, 60x magnification, d) 5CNC0T, 1000x magnification, e) 10CNC0T, 60x magnification, f) 10CNC0T, 1000x magnification.

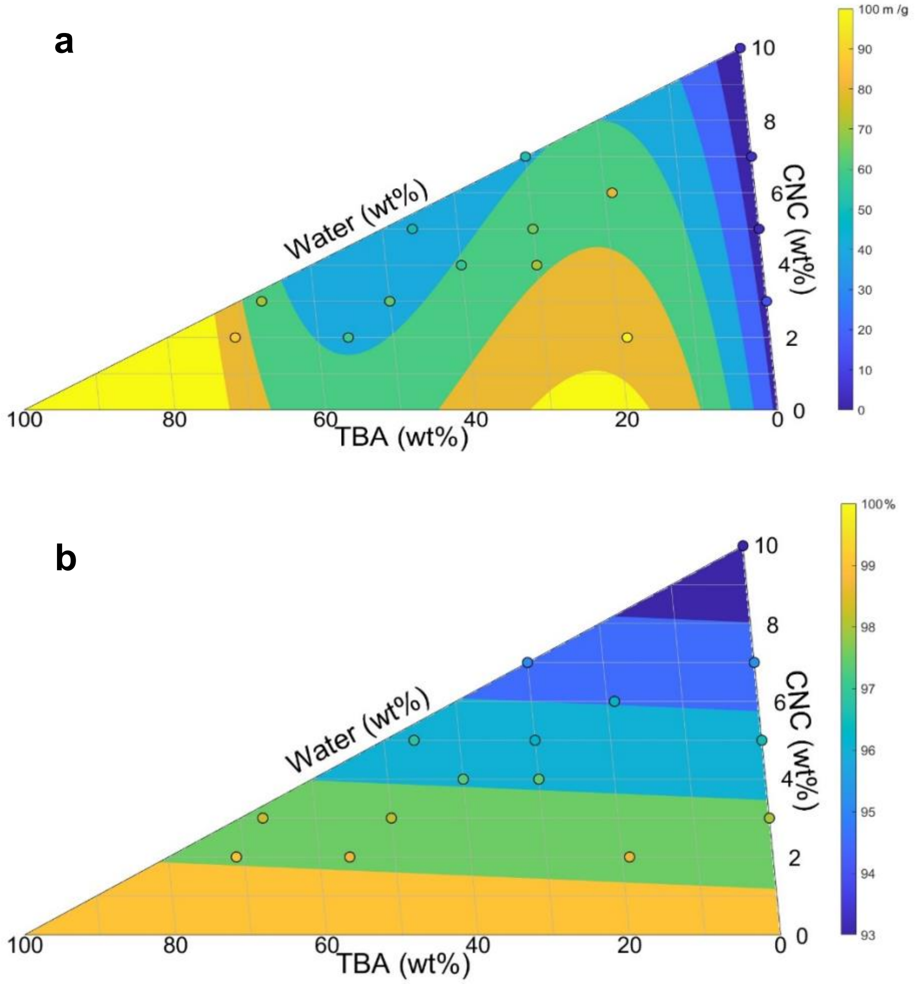

Figure S11. Ternary contour plots of freeze-dried suspensions of CNC, water, and TBA colored according to the value of the a) surface area (m^2/g), b) porosity (%). The contour areas were obtained in JMP by a fitted model, with yellow corresponding to higher values and blue to lower values.

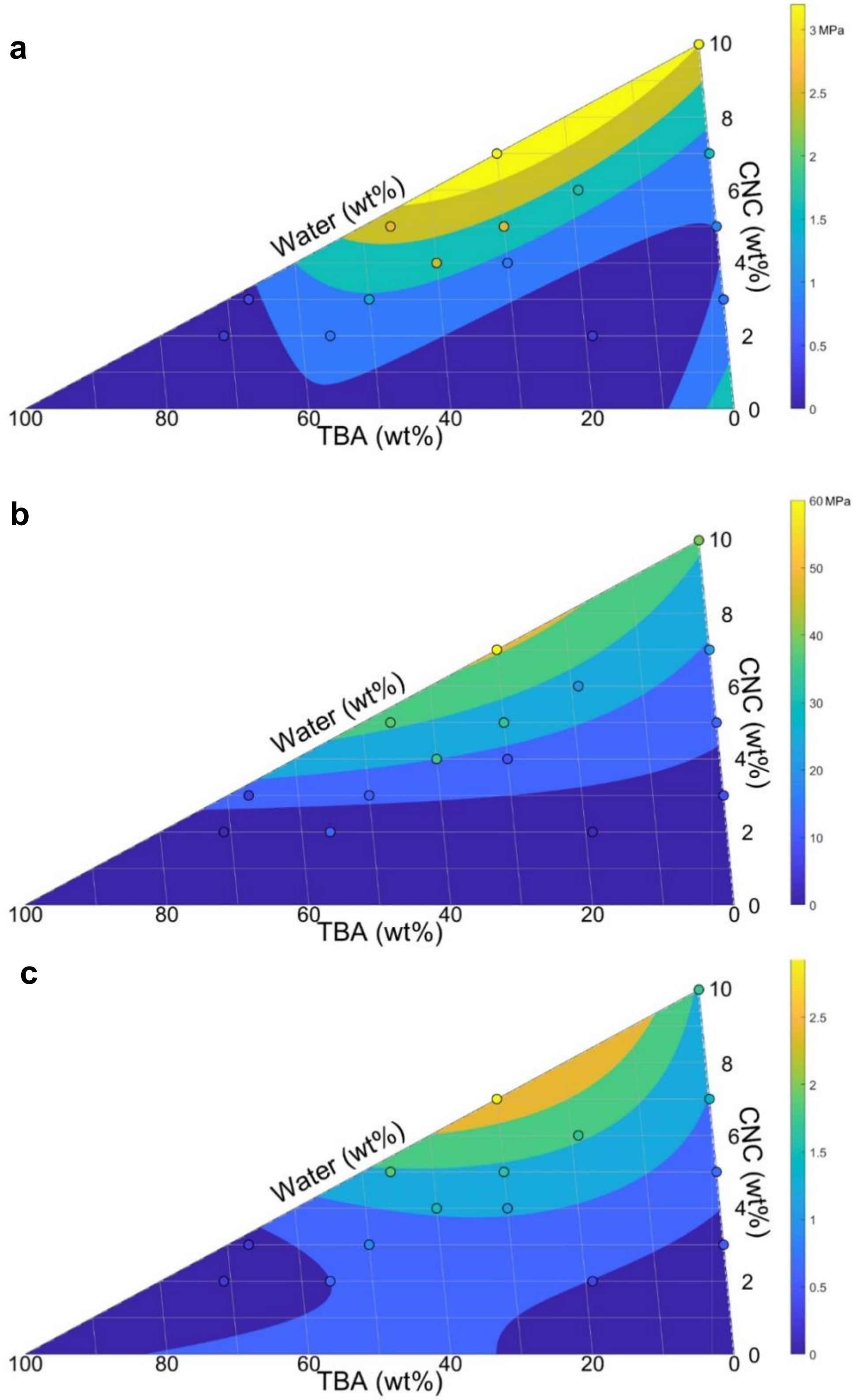

Figure S12. Ternary contour plots depicting compressive properties of freeze-dried suspensions of CNC, water, and TBA colored according to the value of the a) specific energy absorbed (MJ/m^3), b) specific compressive modulus (MPa), c) specific compressive strength (MPa). The contour areas were obtained in JMP by a fitted model, with yellow corresponding to higher values and blue to lower values.

Table S2. Experimental values for surface area, specific modulus, specific energy absorption, and specific compressive strength of freeze-dried solid foams obtained from the suspensions denoted in the first column.

	Surface Area (m^2/g)	Spc. Modulus (MPa)	Spc. Energy Abs. (MJ/m^3)	Spc. Comp. Strength (MPa)
2CNC19T	98	1.9	0.3	0.3
2CNC56T	59	12.5	0.8	0.6
2CNC71T	88	2.1	0.3	0.2
3CNC0T	15	8.6	0.8	0.5
3CNC50T	63	11.4	1.4	0.9
3CNC67T	71	4.9	0.4	0.2
4CNC30T	71	9.0	0.8	1.1
4CNC40T	56	35.6	2.4	1.5
5CNC0T	3	12.2	0.9	0.7
5CNC30T	66	33.8	2.6	1.6
5CNC46T	51	37.7	2.6	1.9
6CNC19T	80	21.0	1.7	1.7
7CNC0T	5	22.0	1.6	1.4
7CNC30T	52	63.4	4.3	3.0
10CNC0T	4	40.0	3.2	1.7

Bibliography

Buchecker, T., Krickl, S., Winkler, R., Grillo, I., Bauduin, P., Touraud, D., Pfitzner, A., & Kunz, W. (2017). The impact of the structuring of hydrotropes in water on the mesoscale solubilisation of a third hydrophobic component. *Physical Chemistry Chemical Physics*, *19*(3), 1806–1816.

Schütz, C., Agthe, M., Fall, A. B., Gordeyeva, K., Guccini, V., Salajková, M., Plivelic, T. S., Lagerwall, J. P. F., Salazar-Alvarez, G., & Bergström, L. (2015). Rod Packing in Chiral Nematic Cellulose Nanocrystal Dispersions Studied by Small-Angle X-ray Scattering and Laser Diffraction. *Langmuir*, *31*(23), 6507–6513.

Svirelis, J., Andersson, J., Stradner, A. & Dahlin, A., 2022. Accurate Correction of the “Bulk Response” in Surface Plasmon Resonance Sensing Provides New Insights on Interactions Involving Lysozyme and Poly (ethylene glycol). *ACS Sensors*, pp. 1175--1182.

De France, Kevin J., Kevin G. Yager, Todd Hoare, Emily D. Cranston. 2016. 'Cooperative Ordering and Kinetics of Cellulose Nanocrystal Alignment in a Magnetic Field', *Langmuir*, 32: 7564-71.
